# A case of mistaken arrhythmogenic identity during pregnancy

**DOI:** 10.1002/ccr3.4561

**Published:** 2021-08-06

**Authors:** Clint Asher, Tessa Thomas, Christopher A. Rinaldi, Gerry Carr‐White

**Affiliations:** ^1^ School of Biomedical Engineering and Imaging Sciences Rayne Institute King’s College London St Thomas Hospital London UK; ^2^ Department of Cardiology Guy’s and St Thomas’ NHS Foundation Trust London UK; ^3^ Department of Acute Medicine Maidstone and Tunbridge Wells NHS Trust Kent UK

**Keywords:** arrhythmogenic right ventricular cardiomyopathy, case report, left ventricular outflow tract, outflow tract tachycardia, pregnancy, right ventricular outflow tract

## Abstract

Atypical LVOT ectopy can present with an RVOT morphology on ECG and differentiation to reveal this focus is in favor of benign idiopathic ventricular ectopy over an arrhythmogenic cardiomyopathy.

## Graphical abstract

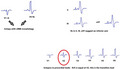

## 1 INTRODUCTION

We present a 31‐year‐old lady with presumed right ventricular outflow tract‐associated ectopy occurring during pregnancy. Through an overview of her presentation, diagnostic workup, and clinical follow up, we highlight the clinical importance of discriminating ectopic morphology in the evaluation of ventricular arrhythmias during pregnancy. A discussion of the case and how the ECG can help differentiate between benign and malignant cardiac substrates in the pregnant population is emphasized in this case report.

Ventricular arrhythmias are a common phenomenon during pregnancy and mostly benign, especially in those without structural heart disease.^1^, ^2^ The largest subgroup of these tends to localize within and around the right and left ventricular outflow tracts; so‐called idiopathic outflow tract tachycardias.^3^, ^4^ They account for the majority of new‐onset ventricular ectopy and can give rise to a significant burden of ectopics during pregnancy, however, these presentations are not usually associated with structural heart disease or sudden cardiac death.^1^, ^5^, ^6^ Treatment is usually conservative, or may involve medication suppression.^5^, ^7^, ^8^ However, in the presence of either ectopy‐related cardiomyopathy; which is a known reversible cause of dilated cardiomyopathy, or in the presence of intrusive cardiac symptoms, curative catheter ablation may be considered.^5^, ^7^, ^8^ The frequent morphology of right ventricular outflow tract (RVOT) ectopy encountered in pregnancy can similarly, occur in pathological disease processes such as arrhythmogenic right ventricular cardiomyopathy (ARVC). This familial disease, characterized by a predisposition to malignant ventricular arrhythmias and an increased risk of sudden cardiac death, clearly needs to be distinguished from the generally benign outflow tract ectopy.^9^

## 2 CASE

A 31‐year‐old teacher of Polish descent presented to the joint cardiac/obstetric clinic at 16‐weeks’ gestation after successful implantation following In vitro fertilization (IVF) and intravenous hormonal supplementation. She was referred after concerns of a possible arrhythmia that arose while under general anaesthesia for egg collection. She had a single unprovoked syncopal episode at 10 weeks and three episodes of pre‐syncope thereafter. She was otherwise well without dyspnoea or palpitations. A 24 hr tape was carried out at her local hospital showing predominantly unifocal ventricular ectopy throughout as singles, bigeminy, and trigeminy, accounting for 17% of her total rhythm, but no sustained arrhythmia.

She had no other medical history of note, was generally fit and well, and prior to pregnancy was exercising frequently with no reported symptoms or indication for cardiac assessment. She was not taking any regular medications. There was no family history of cardiac disease, epilepsy, or sudden cardiac death. Her mother died from a pulmonary embolism during childbirth documented on her death certificate. Her two siblings are both fit and well. She was a non‐smoker, did not drink any alcohol nor consume caffeinated beverages. She did, however, report to take an ephedrine‐based stimulant prior to pregnancy during training sessions.

**FIGURE 1:**
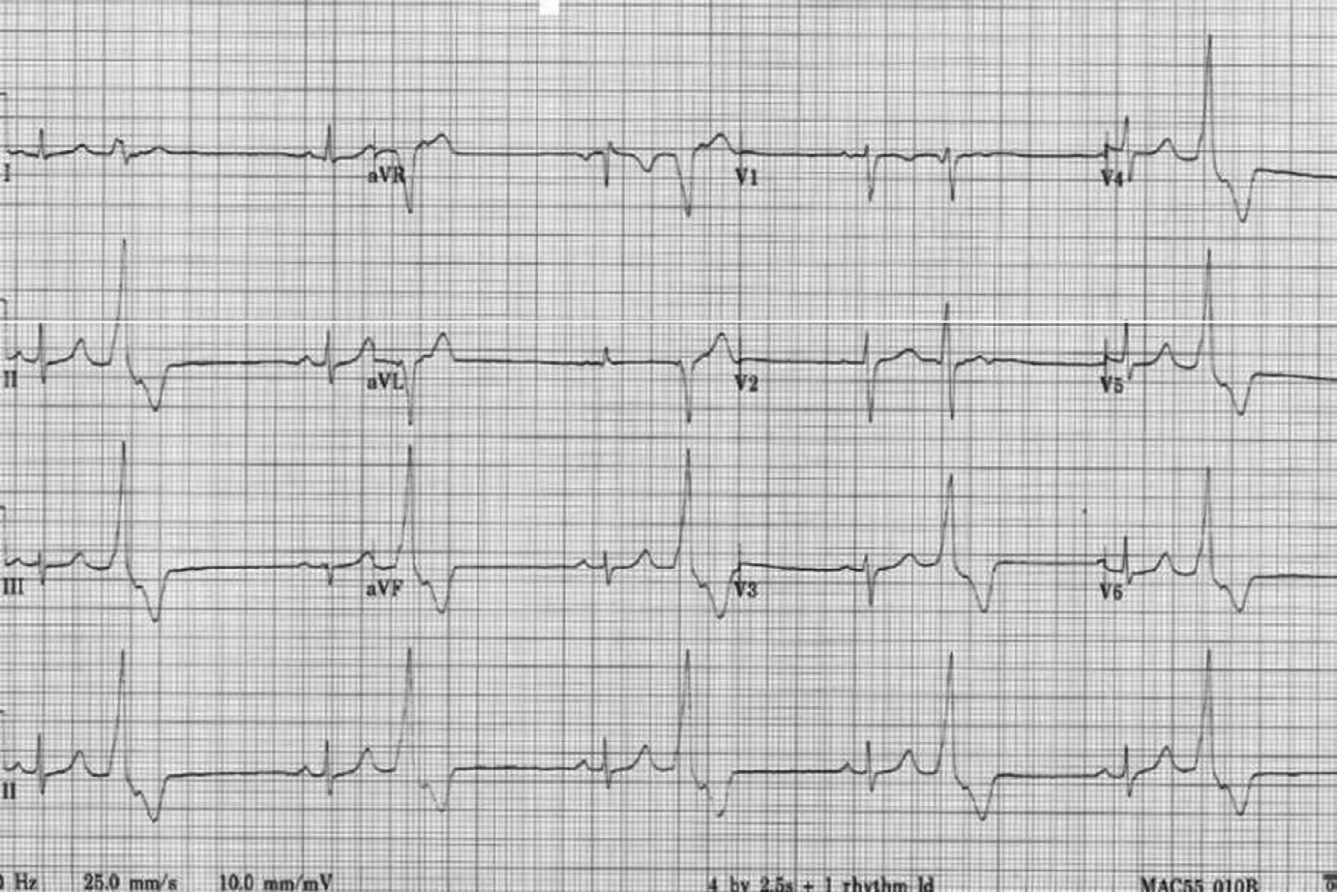
ECG at 16 weeks' pregnant. Bigeminy pattern of ventricular ectopy with a LBBB pattern and inferior axis. LBBB, left bundle branch block

Her examination was unremarkable with a jugular venous pressure of 2 cm and blood pressure of 118/73. 12 lead ECG was evaluated as demonstrating RVOT ectopy on the basis of the left bundle branch (LBBB) morphology with inferior axis (Figure 1). The echocardiogram was unremarkable, with normal biventricular size and systolic function. Routine blood including full blood count, renal function, and thyroid function were within normal limits. Urine analysis was clear. Based on these findings, a cardiac MRI (CMR) was requested to rule out a cardiomyopathy. Her first CMR, without contrast, was at 20 weeks' gestation and demonstrated normal biventricular size but with low‐normal left ventricular (LV) function. LV ejection fraction (LVEF) was 45%–50% in the context of frequent ventricular ectopy. Following an uncomplicated delivery, repeat CMR occurred 5 months later and following gadolinium contrast, there was no evidence of infarction, infiltration, or replacement fibrosis.

Due to the pattern of ectopic burden on 12 lead ECG, 17% burden of ectopics on holter and low‐normal LV function on CMR, she was referred to the inherited cardiac unit for a suspicion of arrhythmogenic “right ventricular” cardiomyopathy (ARVC).

A surveillance clinical review at 12 months' post‐delivery was mostly unremarkable with the occasional awareness of palpitations. Her resting ECG demonstrated a similar pattern of ventricular ectopy, with normal PR, QRS, QTc intervals, and ST‐T wave segments in sinus rhythm. Repeat holter monitoring showed few ventricular ectopics of less than 1% burden of total rhythm. Without receiving medical therapy, her echocardiogram now showed normal biventricular function compared with her MRI post‐birth.

**FIGURE 2:**
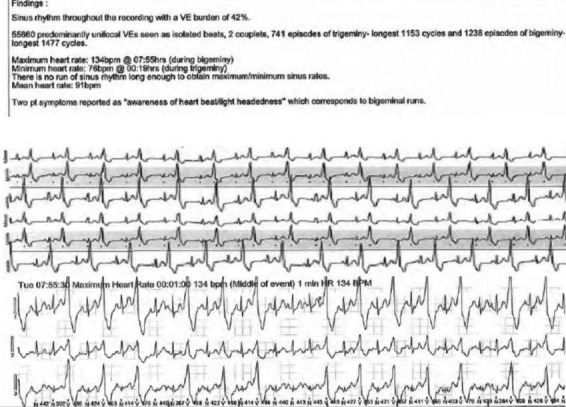
Holter monitor in second pregnancy with frequent ventricular ectopy, burden count of 42% with unifocal expression

She had a second pregnancy with embryo transfer and oral hormonal supplementation 18 months following her first child. At 16 weeks' gestation, her echocardiogram was unremarkable; however, at clinic review, she reported awareness of intermittent palpitations. A holter monitor revealed recurrence of unifocal ventricular ectopy, with a burden of 42% of her total rhythm (Figure 2). There were no sustained arrhythmias. At 28 weeks' gestation, she was reviewed and symptoms were unchanged. LV function on echocardiogram was reassuringly normal, therefore the decision from the clinic was for close monitoring. She delivered spontaneously at term with no complications and remained well postpartum with no symptoms. A repeat holter monitor 6 months postpartum showed few ventricular ectopics including two runs of trigeminy, with a total burden less than 1% of her total rhythm. Her 3rd CMR just over 3 years since her first one was unremarkable with normal biventricular function and with a dedicated protocol showed no subtle/overt features to meet a diagnosis of ARVC.

**FIGURE 3:**
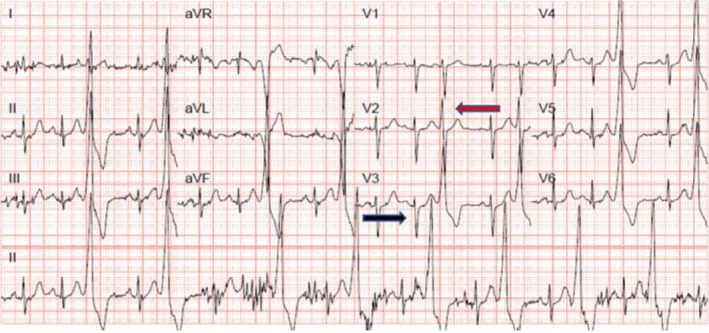
12 lead rhythm strip from holter monitoring. LBBB with the inferior axis. Transition is seen in V2 where R and S wave in the ectopic is equal (red arrow), after which the ectopic becomes more positive by lead V3 in comparison to the sinus QRS – a “disconnect.” Black arrow indicates sinus beat with smaller R and S waves. LBBB, left bundle branch block

In February 2020, she spontaneously conceived and remained well throughout the pregnancy. Due to her history, a holter monitor and echocardiogram were booked at 29 weeks' gestation. Echocardiogram showed low‐normal LV function with LVEF 50%–55% and holter monitor revealed unifocal ventricular ectopic burden of 39%. She was aware of infrequent sensations of palpitations, but was not overtly symptomatic and declined the offer of Bisoprolol. On this occasion, the holter was requested alongside a 12‐lead rhythm strip to review alongside previous ECGs (Figure 3).

Upon review by the inherited cardiac team, it was unanimously agreed that the morphology was consistent throughout all her pregnancies and there was no evidence of progressive features of an ARVC phenotype in the sinus beats on resting ECGs, as well as no structural changes on transthoracic echocardiograms or CMRs. However, given the small “R” waves seen in V1 and an early precordial transition by lead V2, such that the ectopic QRS was more positive than the sinus beat QRS by lead V3, the ectopy origin was refined as coming from an LVOT focus (Figure 3). In conjunction with the other reassuring findings and progress, it was felt this was more in keeping with idiopathic LVOT ectopy, enhanced during the pregnant state and not an underlying inherited cardiomyopathy.

## 3 DISCUSSION

Ventricular ectopic beats are among the most commonly encountered arrhythmias during pregnancy, occurring in more than 50% of pregnant women under investigation for symptoms of palpitations, pre‐syncope, or syncope.^1^, ^10^ Previous studies suggest that these are mostly monomorphic, with equal distribution in their onset over the three trimesters.^10^, ^11^ Although more frequent and troublesome during pregnancy, in those with structurally normal hearts they are considered benign and expected to dissipate without medical intervention or long‐term follow‐up.^4^, ^6^

Pregnancy‐related idiopathic outflow tract ventricular ectopy is a localized subgroup of ectopic beats that are normally attributed to right ventricular outflow tract (RVOT) focus of origin.^11^, ^12^, ^13^ Left ventricular outflow tract (LVOT) arrhythmias are less common in this population, as they typically associate with male gender, older age, hypertension, and left ventricular (LV) dysfunction.^14^, ^15^ Although not fully understood, it seems likely in pregnancy that the various haemodynamic, hormonal, and cardiac changes throughout each trimester influence ventricular arrhythmogenesis.^11^, ^16^, ^17^ Additionally, the necessary increase in cardiac output and stroke volume that contributes to a physiologically dilated heart with increased contractility, may render any pre‐existing substrate sensitive to frequent arrhythmias.^4^, ^16^, ^17^ The dissipation of arrhythmia following delivery suggests the pregnant state has a specific association with the underlying pathophysiology and like other ectopic activity occurring during pregnancy, is usually benign.^11^

In practice, RVOT ventricular ectopy is normally recognized by its specific ECG pattern of LBBB morphology (negative QRS complex in V1), inferior axis, and negativity in the aVL lead (Figure 1). Recognition of this is particularly important as a high burden requires closer evaluation to exclude what could be the expression of an underlying ARVC. This is a familial condition, characterized by progressive fibrofatty infiltration of the ventricular myocardium, with subsequent structural changes and malignant ventricular arrhythmias.^18^, ^19^ During pregnancy, the presence of structural heart disease makes the occurrence of ventricular arrhythmias more significant in terms of adverse risks to mother and baby.^1^, ^12^ Beyond the inherent risks of ARVC in pregnancy, there is a lifelong increased risk of ventricular arrhythmias and early sudden cardiac death.^19^, ^20^ While overt disease is clearly manifested with identifiable changes on ECG and echocardiogram, it is recognized that a “concealed” phase exists with variable expression, whereby patients only demonstrate subtle structural changes and could be asymptomatic prior to a sudden death presentation.^19^, ^21^ During pregnancy, this early phenotype may exhibit ventricular ectopy with multiple morphologies or focal isolated beats with LBBB and inferior axis morphology, thereby mimicking idiopathic RVOT arrhythmias.^19^, ^20^, ^21^, ^22^

Differentiating the two requires meticulous clinical evaluation, particularly taking note of family history, as unlike ARVC, idiopathic outflow tract tachycardias have yet to demonstrate a familial basis. Baseline ECG is the starting point for evaluation of any cardiac disease and of particular benefit in ARVC is repeating this over time to assess for changes reflective of progressive diseases, such as repolarization abnormalities.^18^, ^23^, ^24^

Obtaining a 12 lead ECG concurrently with symptoms would be ideal for evaluation of the ectopic focus. However, as symptoms may be paroxysmal, this is often not achievable and so holter monitoring is often required. It is important to explicitly request for a 12‐lead rhythm strip to review the full ECG signatures of any ectopic beats identified.

Invasive electro‐anatomical mapping has refined much of our understanding of the various sites in and around the outflow tracts that are implicated in ECG patterns of the ventricular ectopy seen.^15^, ^25^, ^26^, ^27^ As such, several differences in ECG characteristics have been reported that should aid differentiation between idiopathic outflow tract tachycardias and ARVC. While LBBB/inferior axis is an overlapping feature, LBBB pattern with the superior or indeterminate axis is more specific for ARVC as it suggests involvement from the RV free wall.^23^, ^28^ Ventricular ectopy that has RBBB morphology is said to originate from an LV focus and is far less common in pregnancy, but may suggest predominant LV myocardial involvement in ARVC.^23^ Finally, there are atypical presentations of ventricular ectopy that arise from the LVOT and aortic cusp producing a similar morphology to RVOT focus arrhythmias, as was suggested in our case.^29^, ^30^ These are important to unearth, as these focal areas generally imply benign disease and can be managed as other benign ectopy encountered in pregnancy.^31^ The mechanism of frequent ventricular ectopy in these cases may differ from those with a typical RVOT focus, and the triggers may be more specific to hormonal fluctuations.^32^ This reasoning is proposed with our patient as her high burden of ectopy only presented during the pregnant state and following fertility hormonal supplementation, with no evidence to suggest prior occurrences. Her symptoms and ectopy also resolved following each of her pregnancies.

ECG differentiation from ARVC is possible first by evaluation of QRS duration in lead 1, as a duration greater than 120ms has been reported to have a sensitivity of 88%.^25^ Precordial transition references the lead where R and S wave amplitude are equal and this usually occurs late in ARVC, around V5‐V6.

**FIGURE 4:**
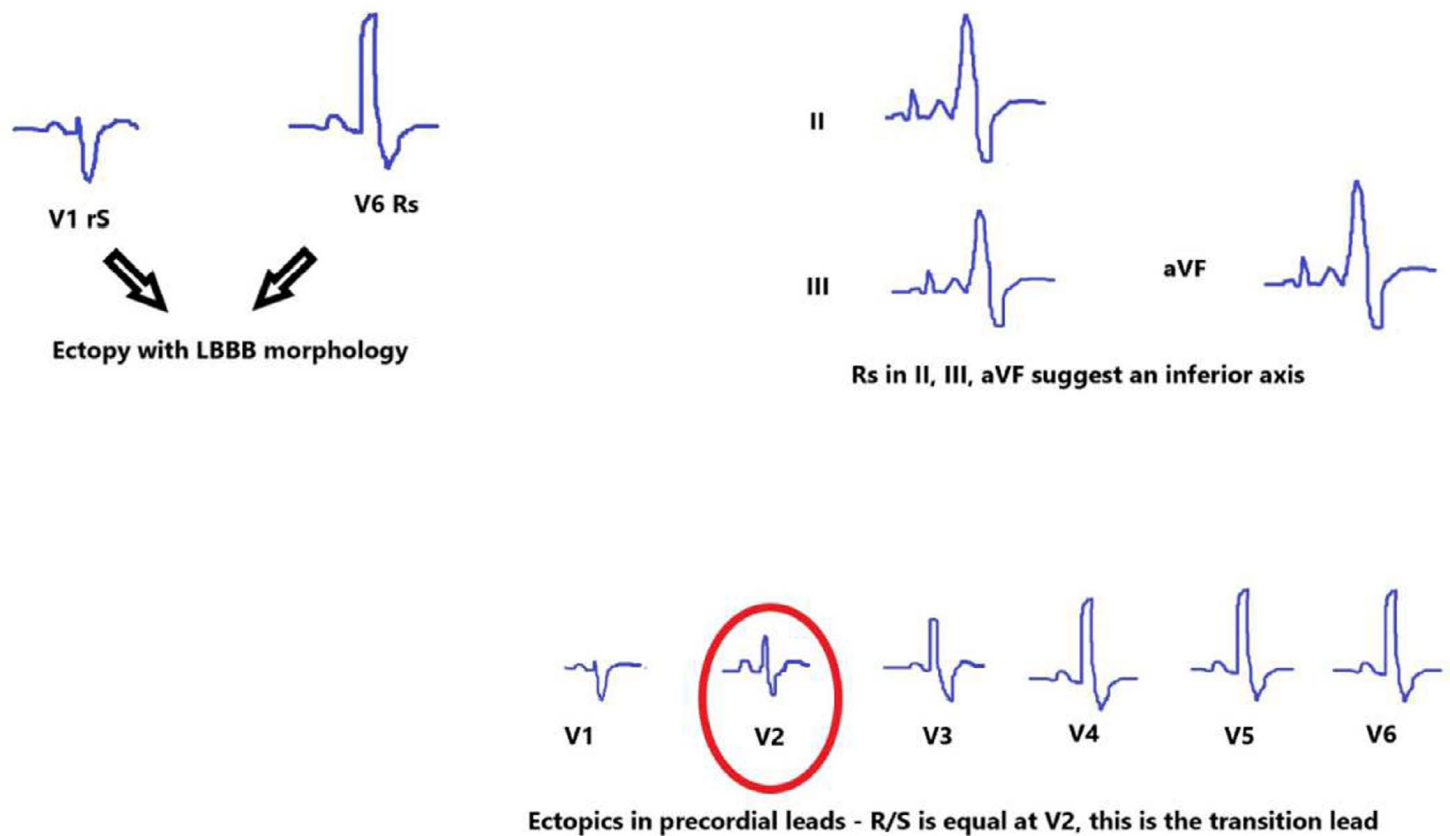
Schematic outlining main features of atypical LVOT focus. LBBB morphology was identified by a review of ectopic morphology in V1 and V6. Positive ectopy in leads II, III, aVF imply an inferior axis. Review of the chest (precordial) leads show R/S wave is equal in V2, this is the transition point, and should be greater than the R/S wave in the sinus beat. LBBB, left bundle branch block

Identification of the bundle branch pattern in V1 and the precordial transition are consistent features described in all the published algorithms to have the highest sensitivity and specificity in differentiating RVOT ectopy from atypical LVOT presentations that also produce ectopics with LBBB/inferior axis.^26^, ^27^, ^30^ As was seen in our case, the presence of a small r‐wave in V1 and earlier precordial transition in ectopic beat compared with sinus beat by lead V2, is an independent predictor for an LVOT focus.^27^, ^29^, ^33^, ^34^ As this ECG signature from an LVOT focus has not been described in ARVC, even in those with subtle structural features, this is an additional, indispensable tool in the evaluation of frequent ectopy with RVOT morphology in the pregnant patient.^21^, ^23^, ^35^ These features are summarized in Figure 4.

Evaluation of ECG characteristics should be combined with anatomical information to facilitate the exclusion of structural disease. In most cases, CMR is the established gold standard for ARVC assessment given the additional cross‐sectional images and enhanced field of view.^28^ As ARVC is a progressive disease, serial assessment is warranted to detect any early manifestations, particularly in those imaged while pregnant when contrast enhancement is contraindicated.

## 4 CONCLUSION

This case report documents the evaluation of ventricular ectopy arising in pregnancy. While often a benign phenomenon requiring reassurance alone, contemporary evaluation of ventricular ectopy that is frequent with RVOT morphology should include assessment of ECG characteristics combined with pertinent family history and imaging in order to differentiate idiopathic outflow tract ectopy from malignant phenotypes such as ARVC.

### 4.1 Limitations

The main limitation is that for our patient there was no invasive electro‐anatomical mapping to correlate the source of ectopic focus, however, this was not clinically indicated due to the tolerability of symptoms, resolution post‐delivery, and absence of sustained structural heart disease.

### 4.2 Learning points

- Ventricular arrhythmias are common during pregnancy and are mostly benign, not requiring specific treatment.
- Idiopathic outflow tract tachycardias are an important subgroup that the represent the commonest form of idiopathic VT in pregnancy with an RVOT focus seen more frequently than LVOT focus.
- Predicting the site of outflow tract arrhythmias has important implications during pregnancy.
- Early ARVC may mimic an idiopathic phenotype as it often presents with frequent ventricular ectopy of RVOT morphology with little or no other associated features.
- Early ARVC is associated with malignant ventricular arrhythmias and risk of sudden cardiac death, and is important to exclude in this vulnerable group of patients.
- Atypical LVOT ectopy can also present with an RVOT morphology on ECG and differentiation to reveal this focus is in favor of benign idiopathic ventricular ectopy over ARVC.
- Careful ECG differentiation should be included in the multiparametric clinical approach to assessing ventricular arrhythmias in pregnancy.

## CONFLICTS OF INTEREST

None declared.

## AUTHOR CONTRIBUTIONS

GCW was the lead clinician for the patient, conceived the case report and was involved in writing and formatting the manuscript. CA was a member of the team involved in the patient's care, obtained patient permission, and was involved in writing, editing, and formatting the manuscript. TT was involved in writing, editing, and formatting the manuscript. CAR was involved in the critical review and editing of the manuscript.

## ETHICAL APPROVAL

The authors confirm that informed consent for submission and publication of this case report including image(s) and associated text has been obtained from the patient.

## DATA AVAILABILITY STATEMENT

Data sharing not applicable to this article as no datasets were generated or analysed during the current study.
